# New Radionuclides and Technological Advances in SPECT and PET Scanners

**DOI:** 10.3390/cancers13246183

**Published:** 2021-12-08

**Authors:** Nicholas P. van der Meulen, Klaus Strobel, Thiago Viana Miranda Lima

**Affiliations:** 1 Center for Radiopharmaceutical Sciences ETH-PSI-USZ, Paul Scherrer Institute, 5232 Villigen, Switzerland; 2 Laboratory of Radiochemistry, Paul Scherrer Institute, 5232 Villigen, Switzerland; 3 Department of Radiology and Nuclear Medicine, Luzerner Kantonsspital, 6000 Luzern, Switzerland; klaus.strobel@luks.ch; 4 Institute of Radiation Physics, Lausanne University Hospital, University of Lausanne, 1007 Lausanne, Switzerland

**Keywords:** nuclear medicine, PET, SPECT, radionuclide, development

## Abstract

**Simple Summary:**

Advances in nuclear medicine are made by technological and radionuclide improvements. Throughout nuclear medicine’s history, these advances were often intertwined and complementary based on different clinical questions, availability and need. This paper covers some of these developments in radionuclides and instrumentation.

**Abstract:**

Developments throughout the history of nuclear medicine have involved improvements in both instrumentation and radionuclides, which have been intertwined. Instrumentation developments always occurred during the search to improving devices’ sensitivity and included advances in detector technology (with the introduction of cadmium zinc telluride and digital Positron Emission Tomography—PET-devices with silicon photomultipliers), design (total body PET) and configuration (ring-shaped, Single-Photon Emission Computed Tomography (SPECT), Compton camera). In the field of radionuclide development, we observed the continual changing of clinically used radionuclides, which is sometimes influenced by instrumentation technology but also driven by availability, patient safety and clinical questions. Some areas, such as tumour imaging, have faced challenges when changing radionuclides based on availability, when this produced undesirable clinical findings with the introduction of unclear focal uptakes and unspecific uptakes. On the other end of spectrum, further developments of PET technology have seen a resurgence in its use in nuclear cardiology, with rubidium-82 from strontium-82/rubidium-82 generators being the radionuclide of choice, moving away from SPECT nuclides thallium-201 and technetium-99m. These continuing improvements in both instrumentation and radionuclide development have helped the growth of nuclear medicine and its importance in the ever-evolving range of patient care options.

## 1. Introduction

**Figure 1:**
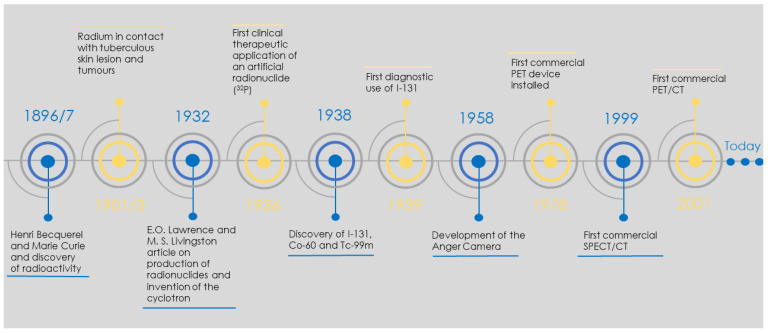
Concise nuclear medicine historical timeline which covers the most notable events. Timeline based on SNMMI historical timeline [1].

The world of nuclear medicine has been dynamic and fast-paced since the discovery of radioactivity by Marie and Pierre Curie in 1897 and the development of the Anger camera in the late 1950s. The period from the discovery of radioactivity to its medical use covered a mere decade. This was followed by developments in radionuclide production, pharmacokinetics and molecular radiotherapy. According to the Society of Nuclear Medicine and Molecular Imaging’s (SNMMI) historical timeline [1], the advancement of nuclear medicine was always linked to the almost symbiotic evolution of radionuclides, radiopharmaceuticals and instrumentation, as shown in Figure 1.

This evolution allowed nuclear medicine to constantly challenge other imaging and therapeutic modalities, as shown recently by Hirmas et al. [2] where they demonstrated the superiority of Ga-68-labelled PSMA over conventionally used Computed Tomography (CT) in the diagnosis and treatment management of hepatocellular carcinoma (HCC) patients. This was further demonstrated by the phase 2 randomized trial DOSISPHERE-01 that showed the superiority of Y-90 radioembolization over conventional chemoembolization, which subsequently improved overall patient survival in the treatment of HCC [3].

**Figure 2:**
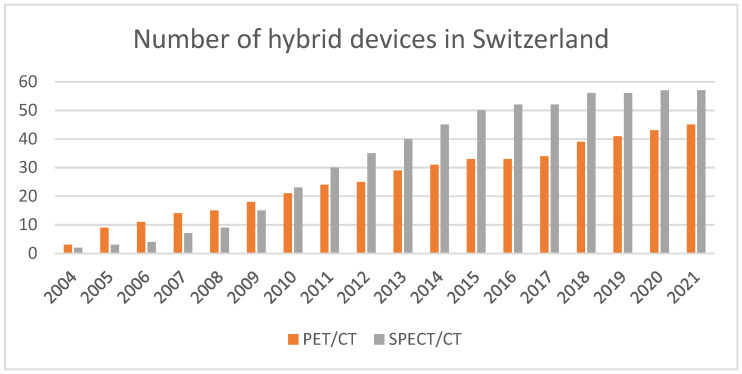
Number of PET/CT and SPECT/CT in Switzerland. Stand-alone SPECT devices were not included, since local license regulations only account for holding, manipulation and disposal of radionuclides rather than devices; one license can include several stand-alone devices. CT devices, including hybrid devices, require single licenses.

With the introduction of Single-Photon Emission Computed Tomography (SPECT), Positron Emission Tomography (PET) and, later, hybrid imaging including SPECT/CT, PET/CT and PET/MRI, nuclear medicine instrumentation has progressed greatly over the last two decades [4]. The first commercially available SPECT/CT was launched in 1999 [5] and the first PET/CT in 2001 [6] and, since then, there has been a steady increase in device numbers in Switzerland alone, as shown in Figure 2. This transition made the link between the powerful physiological information present in nuclear medicine examinations and the anatomical structures visible in CT and MRI scans. The more recent clinical translation of theragnostics (where one can treat what one images) beyond thyroid treatment has triggered a dramatic transformation of the nuclear medical field [4].

Overall, the development of radionuclides and radiopharmaceuticals is often driven by instrumentation developments. In this work, the impacts of these evolutions and the future trends in nuclear medicine are presented.

## 2. Instrumentation Developments

The concepts of both PET and SPECT designs are not much different from the initial designs by Anger in the 1950s [7], where devices had a scintillator crystal coupled to photomultipliers and electronics. With advances in computer power, a huge number of changes was observed, which boosted improvements in the electronic components and reconstructions and applied corrections for both devices. This was translated into developments and improvements in reconstruction methods and corrections, such as point-spread-function (PSF). The benefits [8] and challenges [9] of using these improvements are well described in the literature. Accompanying the computational evolutions were advancements in the fields of scintillation material and detector design.

Nowadays, there is a greater translation between PET and SPECT for different clinical questions. There are inevitable technical differences between the devices, which influence the choice of one device over the other, such as reduced scan times and, subsequently, patient comfort, availability and improved sensitivity and spatial resolution. Additionally, with the evolution of instrumentation, we have observed the resurrection of radionuclides that were previously out of vogue.

### 2.1. The Detector Revolution

**Figure 3:**
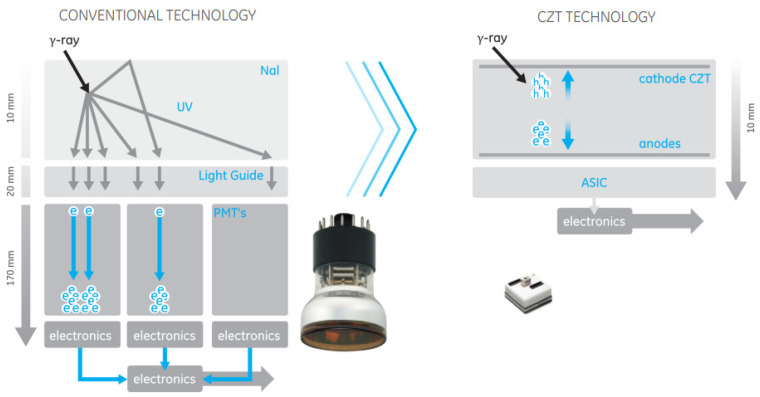
Comparison between detector arrangement of the more conventional NaI detector versus CZT crystal and sensors. Image from General Electric (GE) CZT detector, courtesy of GE Healthcare.

A technique that has been introduced to nuclear medicine over the years was the use of more efficient detectors with better physical characteristics for imaging, for example, the transition from BGO to L(YSO) in PET that allowed the introduction of time-of-flight in PET reconstruction [8]. This transition took a bit longer to occur for the gamma (Anger) camera and arrived with the introduction of semiconductor detectors. The detector measures the electric charge by collecting the electrons/holes generated when radiation enters the crystal and interacts with it, as seen in Figure 3. One initial limitation of this type of detector was that it was necessary to keep them cold to reduce thermal noise [10]. This was subsequently overcome with the development of cadmium–zinc–telluride (CZT) crystals [10]. Due to the large size of the band gap in the CZT detector, it can be used at room temperature, and the high atomic number of CZT assists in efficient photoelectric absorption, leading to an improved system sensitivity compared with that of the sodium iodine (NaI) detector [11].

**Figure 4:**
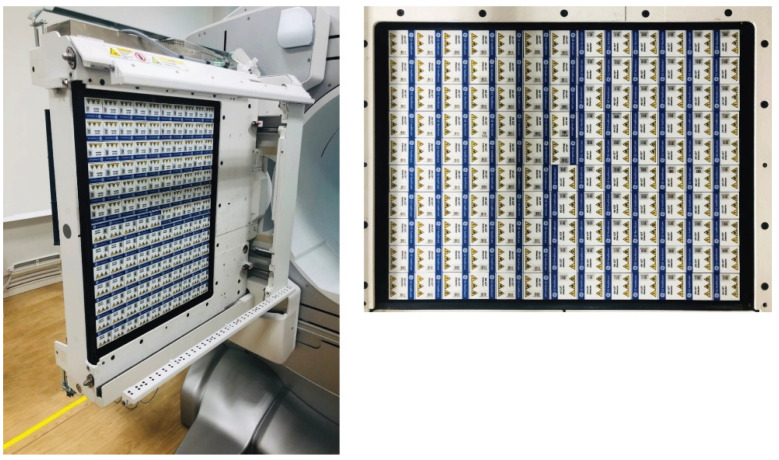
GE Discovery: CZT SPECT device. Figure from [11]. Representation of the detector arrangement for a general-purpose CZT device.

The first clinical CZT devices that were introduced were anatomically specific for cardiac examinations [12,13,14,15,16,17,18,19]. The first example of a general-purpose CZT device (Figure 4), coupled with a Computed Tomography (CT), was introduced by General Electric (GE) Healthcare. Several clinical studies using this device have been reported, covering different aspects including the reduced radiation burden due to higher sensitivity, verification of dual-radionuclide imaging and short time acquisition using Tc-99m and I-123 in myocardial blood flow tests [20,21].

**Figure 5:**
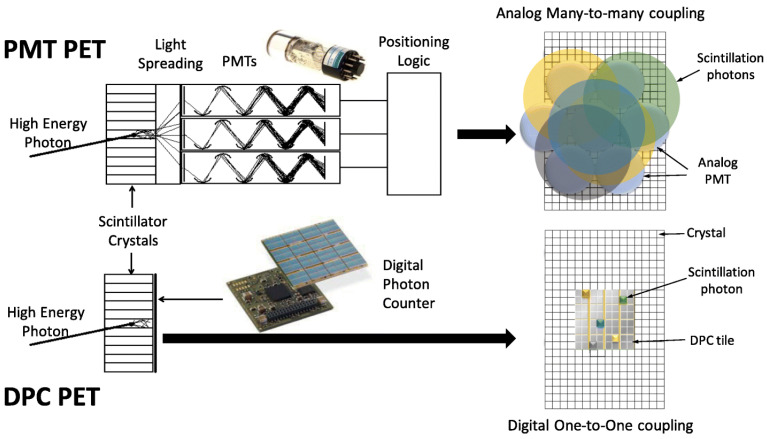
Example of PET-detector block: conventional photomultiplier tube (PMT) and silicone photomultiplier (DPC). The main improvements are seen in the use of smaller SiPM, with full coverage of the crystal possible, in addition to faster electronics [23].

The last decade has also seen a huge evolution in PET devices. Improvements in corrections (including TOF, scatter correction and point-spread-function) were followed by advances in detector technology. The latest generation of PET/CTs, popularly known as digital PET [22], included the introduction of silicone-photomultipliers (SiPM) in the process of identifying the signal obtained from radioactivity emissions in an image (Figure 5).

**Figure 6:**
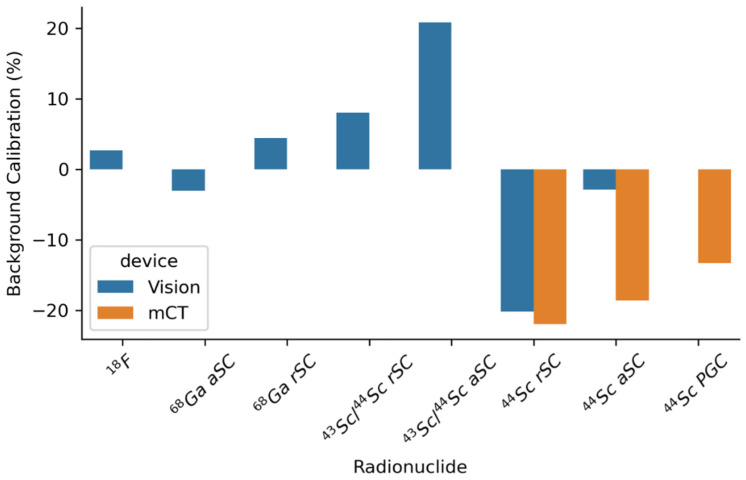
Comparison of the impact of different scatter corrections on background calibration, as presented by Lima et al. [24], for two generations of devices. The main difference is the improvement in the quantification observed between devices due to higher sensitivity and reduced energy windowing, as well as the superior time-of-flight on the newer-generation device.

The development of highly compact solid-state devices to convert scintillation photons to electrical signals has led to improvements in detector compactness, accuracy and timing. These, and similar devices, have also enabled imaging using high magnetic fields in PET/MRI, which is not possible with standard photomultipliers [4]. These improvements in both detector sensitivity plus time-of-flight and reduced energy windowing have had a high impact on the quantification of non-pure positron emitters [24]. Figure 6 shows the improvement in quantitative accuracy between two generations of PET/CT devices for Sc-44, which has a high energy prompt gamma that influences the scatter correction of the system.

### 2.2. New Arrangements

**Figure 7:**
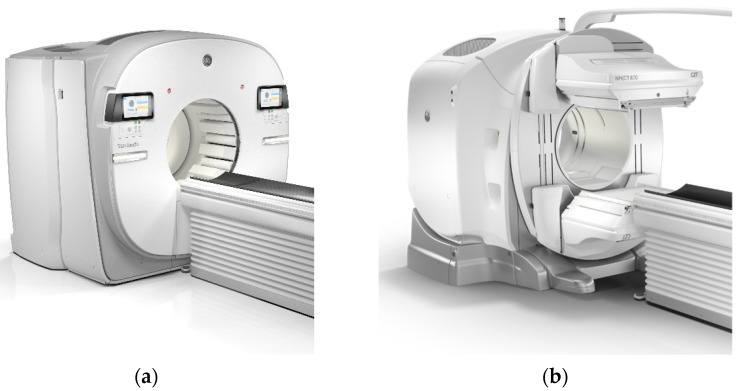
Ring-shaped SPECT: (**a**) StarGuide (GE Healthcare, Chicago, IL, USA) side-by-side with the conventional multi-head SPECT design, (**b**) NM/CT 870 (GE Healthcare, Chicago, IL, USA). Courtesy of GE Healthcare.

Nuclear medicine devices have evolved following the need to improve image quality, quantification aspects and clinical information. This saw the translation from purely planar devices to tomographic devices capable of producing 3- and 4-dimensional images. Lately, apart from the detector revolution presented before, there is also a leap towards modified designs (Figure 7).

This was more evident in SPECT, with the introduction of specific clinical designs targeting dedicated clinical questions, such as the development of cardiac-specific devices as described before; however, the general-purpose device continues to have the well-known design with a gantry and multiple detector heads.

Spectrum Dynamics (Shanghai, China) and GE Healthcare (Chicago, IL, USA) presented a different approach with their Veriton and StarGuide systems, respectively. While conventional SPECT devices have detector heads attached to a rotating gantry, and their tomography image acquisition is obtained by positioning the heads at different angles, the ring-shaped SPECT (Figure 7) uses the same design as PET. The detectors are positioned in fixed positions (12 dedicated positions for both devices) around the ring, and the acquisition is obtained using these fixed positions. The main change is seen by moving smaller individual detectors such that these systems are able to reduce the distance to the required anatomical location, producing images with improved detail [25].

Most recently, long-axial field-of-view scanners with SiPM detection systems have been introduced, with Badawi et al. reporting the first clinical experiences with a 194-cm FOV scanner (uExplorer, United Imaging Healthcare Co, Shanghai, China) [26], which provided substantially improved sensitivity when compared with previous-generation standard axial field-of-view systems [27]. Additionally, the first Biograph Vision Quadra PET/CT system worldwide (Siemens Healthineers, Knoxville, TN, USA), with a FOV of 106 cm, was installed at the Department of Nuclear Medicine, Inselspital, Bern, Switzerland. Preliminary assessments of this scanner’s characteristics reveal a sensitivity of 174 cps/kBq and a time of flight (TOF) resolution of 219 ps in ultra-high sensitivity mode [28], which is 74% higher than a PET device with similar technology. A standard axial of a field-of-view Biograph Vision 600 (Siemens Healthineers, Knoxville, TN, USA) system has an axial FOV of 26.3 cm, 214 ps and 100 cps/kBq [29].

**Figure 8:**
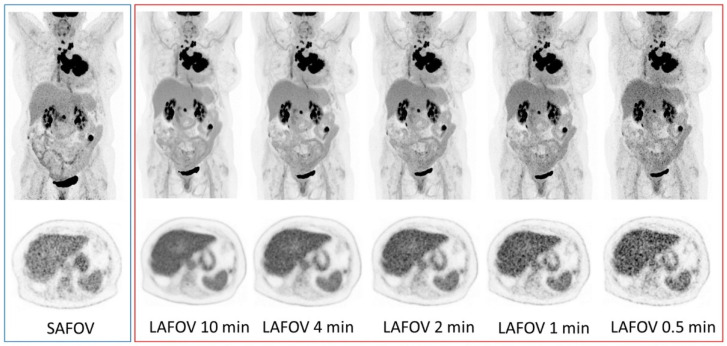
Representative maximum intensity projection (MIP, top row) and axial PET images (bottom row) of a 57-year-old female with non-small-cell lung cancer. Presented are images for the standard axial field-of-view acquisitions on the left (blue margin) and the large axial field-of-view for 10-, 4-, 2-, 1- and 0.5-min acquisitions (right, red margin). For reference, the PET window was set to 0 to 8.5 SUV. Image from Alberts et al.; Eur. J. Nucl. Med. Mol. Imaging [27].

Figure 8 shows the direct comparison between two similar technology systems with the size of the axial field of view being the only difference. Since the gain in sensitivity translates into improved signal-to-noise ratio, users have the choice to reduce the patient radiation burden by reducing the injected activity or to improve patient experience by reducing acquisition times while keeping image quality constant. As pointed out by the authors of [27], state-of-the-art standard axial field-of-view PET has already shown improved lesion quantification, improved diagnostic certainty and inter-reader reliability compared to previous-generation devices. The open question for the years to come is where (outside dose reduction and scan time) these long axial field-of-view PET devices will find their clinical niche.

**Figure 9:**
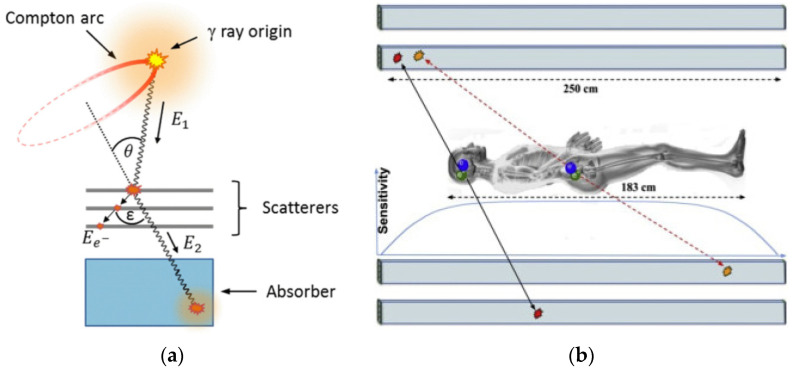
(**a**) The principle of gamma-ray tracing in a Compton camera (Figure reproduced with permission from Aldawood et al. [30]). (**b**) J-PET design with double layer of long plastic scintillators (Figure modified with permission from Pawel et al. [31]).

In addition to extending the axial field-of-view PET, comprised of multiple detector rings and ring-shaped SPECT, there are currently developments in the design of new, improved PET and SPECT-like devices. Current clinical PET and SPECT devices produce imaging by absorbing gamma photons in their scintillator crystals. A different approach proposed by some groups is to use multiple layers of detectors, thereby generating images from both absorbed gamma as well as the interactions of a stack of detectors [30], as shown in Figure 9. One such proposed design (J-PET), in addition to benefitting from this multi-layer detector, also uses long plastic scintillators (across the entire axial field of view and an order of magnitude cheaper than crystals) coupled with photomultipliers and electronics at its ends, instead of having multiple detector rings (Figure 9) [31].

### 2.3. Modality Variation

Selective internal radiation therapy (SIRT) is a radionuclide therapy aimed to treat liver tumours or metastases in patients who are not eligible or cannot tolerate transarterial chemoembolization (TACE). Yttrium-90 resin or glass microspheres are injected into the liver via a catheter during a SIRT interventional procedure. The majority of the yttrium-90 microspheres are transported to the tumour due to its increased vascularization and, subsequently, high blood flow. Therefore, a higher dose is deposited in the tumour than in the healthy liver tissue [32].

Yttrium-90 is mainly a β^−^ emitter, with a very small branching ratio for positron production. In 0.003186% of the decay, there will be the emission of an e^+^/e^−^ pair at 1.76 MeV. As the transition energy is 1.76 MeV, 738 keV remains as kinetic energy to be split between the electron and the positron to conserve the null momentum.

Initially, two main randomized control trials (SARAH [33] and SIRverNIB [34]) evaluated SIRT impact in comparison to its non-radioactive competitor. Both trials showed the primary endpoint was not met, i.e., they did not find significant improvement in overall patient survival. However, they discovered that SIRT is a better-tolerated treatment with reduced frequency and severity of side effects [33,34], providing a better quality of life [33]. SIRT also improved overall and liver progression-free survival and time to progression in the SIRveNIB study [34]. Still, SIRT dosimetry considerations should be studied further [35], especially since the planned activity and absorbed dose in both trials were based on the body surface area (BSA), i.e., no endpoints regarding tumour- and liver-absorbed doses were planned [35].

A recent publication [3], based on a phase 2 randomized clinical trial, attempted to overcome these previous works’ limitations and looked into the impact of personalized dosimetry in SIRT. They found that personalized dosimetry significantly improved the objective response rate in patients with locally advanced hepatocellular carcinoma compared with standard dosimetry. This study suggests that personalized dosimetry is likely to improve clinical practice outcomes and should be used in future trials of selective internal radiation therapy.

One challenging aspect with Y-90 dosimetry is imaging quantification. Y-90 SPECT imaging is based on collecting Bremsstrahlung photons generated as a result of the interaction between the electrons originating from the β^−^ decay and the tissue [36]. Due to this indirect imaging, various disadvantages are observed, such as its low spatial resolution (up to 15 mm) that depends on energy window width, collimator choice and image processing [36]; attenuation correction, since it depends on the density of the objects through which the photon passes, as well as the photon’s energy [37]; and overall quantification capability translating into an inaccurate dose-response analysis, despite the possible compensation techniques for attenuation, scatter and collimator detector response [38,39].

These disadvantages aided the translation into Y-90 PET imaging, where quantification is obtained. The limited positron-branching ratio is overcome with the improved sensitivity of new digital devices and TOF technology, which enables the acquisition of the annihilation photons, thus obtaining improved resolution images of the microsphere biodistribution. Moreover, Y-90 PET images have been proven to be suitable for quantification and, thus, potential use for post-SIRT dosimetry [36]. A recently published work, written by an international collaboration of experts, strongly recommends the use of Y-90 PET for post-therapy evaluation, due to its superior quantification capabilities in comparison to Bremsstrahlung SPECT [40].

## 3. Radionuclide Development

The hunt for the perfect radionuclide in nuclear medicine imaging and therapy is ever changing. The radionuclide selection criteria depend on the physical data of the radionuclide in question (half-life, particulate emissions, gamma-ray emissions), production method, separation chemistry, labelling chemistry and biological behaviour [41]. In general, for imaging purposes, a shorter half-life is desired, as it would aid in reducing the patient radiation burden, but not so short that would limit its use (from production, through labelling, delivery, up-take time and imaging). It is also important for the radionuclide emission to be able to reach the detector. For therapies, physical half-life should be comparable to, or longer than, the biological half-life of the carrier molecule or elemental radionuclide in question [41]. The radionuclide’s short-range radiation should allow crossfire to partially overcome uptake heterogeneity in small tumours. Sometimes, the therapeutic radionuclide emits some imageable photons, which can allow a low-dose imaging study of dosimetry calculation prior to the therapeutic administration [41].

When irradiating target material towards the production of a specific radionuclide, only a small proportion thereof undergoes the nuclear reaction from the irradiation. The final product, to be used for medical application, must be treated in such a way that it is free of contaminants that can compete with the radiometal for the labelling of biologically active molecules used in nuclear medicine. This makes radionuclide development particularly challenging, as one needs to determine the optimal irradiation conditions of the target material, as well as establish a fast and highly efficient post-irradiation chemical separation procedure. To date, the most effective means of achieving the goal of separating high-radiation-dose radionuclides from side products and target material has involved the use of ion exchange and extraction resins. Typically, after irradiation of the target, the target is removed from the irradiation station and, in many cases, also from its encapsulation (or quartz ampoule in the case of neutron irradiation) after which it is dissolved with an appropriate solution. This solution may be adjusted towards the desired, chosen, optimum conditions required for the most effective separation using the ion exchange resin in question. Should one wish to obtain a high activity per volume product, the eluted fraction from the ion exchange resin, containing the desired purified radionuclide, can be passed through an extraction resin column for concentration purposes, from which it can be eluted in a small volume [42].

Lastly, an important aspect of radionuclide development is the constant search for improvements in theragnostics, where a strong link between imaging and therapy is needed. With some of the current clinical success examples, such as the Ga-68/Lu-177 combination, there have been several reports about the impact of using radionuclides with different coordination chemistry to create different chelator-radiometal complexes, leading to different uptake patterns between diagnostic and therapeutic radioligands [43,44,45]. Ideally, radioisotopes of the same element are desired to ensure the true concept of theragnostics, in that what one images is what one treats.

### 3.1. Drive for Radionuclide Development

The initial success story of theragnostics in nuclear medicine began with iodine radioisotopes (I-131, I-123 and I-124). Radionuclides of the same element allowed the preparation of chemically identical radiopharmaceuticals for diagnosis and therapy, enabling the concept of radiotheragnostics in the truest sense [46]. In this regard, scandium and terbium are of particular interest, as they present several radioisotopes which may be of value for clinical translation [46,47,48].

Scandium has attracted the attention of researchers and nuclear physicians alike, due to the existence of matched radioisotopes which could have a possible theragnostic application [44,46,47,48,49,50]. Sc-43 and Sc-44 are promising for PET imaging, with image quality comparable to the more common clinically used radionuclides, such as F-18 and Ga-68 [24,51]. Sc-47 is a β^–^ emitter suitable for therapeutic purposes and also produces γ-ray emissions that are useful for SPECT imaging. The application of Sc-43/Sc-44 (T_1/2_ = 3.9 and 4.0 h, respectively) for PET would be advantageous when comparing it to the most-employed radiometal currently, Ga-68 (T1/2 = 68 min); the almost four-fold longer half-lives of Sc-43/Sc-44 would enable the shipment of Sc-43/Sc-44-radiopharmaceuticals to distant PET centres [46]. In addition, images could be acquired over longer periods. Finally, the stable co-ordination of scandium with 1,4,7,10-tetraazacyclododecane-1,4,7,10-tetraacetic acid (DOTA) can allow the application of the same targeting agents as are subsequently used for therapeutic applications [46,52]. Sc-43/Sc-44 may, therefore, be employed for diagnosis, as well as for planning and monitoring targeted radionuclide therapy with Lu-177 and Y-90. The exact matched therapeutic counterpart Sc-47 would be even more appealing, as it can enable the concept of using chemically identical radiopharmaceuticals with the same kinetic properties for diagnosis and therapy [46]. Currently, Sc-47 cannot be produced in the quantity required for clinical application.

Terbium is unique in that it represents radioisotopes for all four modalities in nuclear medicine [46,53]. Tb-155 (T_1/2_ = 5.3 d) emits γ-radiation for SPECT imaging, and Tb-152 (T_1/2_ = 17.5 h) decays by the emission of positrons that are useful for PET. The decay of Tb-161 (T_1/2_ = 6.9 d) is characterized by the emission of low-energy β^—^ particles and γ-rays, similar to Lu-177, but, additionally, comprises a significant number of Auger/conversion electrons (~12 e^−^/decay). It is, therefore, a promising candidate for therapeutic purposes [46,54,55,56]. Auger electron emitters have very attractive properties for cancer therapy, since their nanometre–micrometre range results in a high LET, which is potent for causing lethal damage in cancer cells [57]. Tb-149 (T_1/2_ = 4.1 h) decays by the emission of α-particles, potentially allowing its use for α-therapy [46,58]. Since terbium belongs to the group of lanthanides, stable coordination is feasible with DOTA, a macrocyclic chelator that is commonly used for the chelation of Lu-177. The production of Tb radioisotopes and their chemical (lanthanide) separation are not trivial processes, which is why Tb radioisotopes have not yet been translated to a clinical routine [46].

**Figure 10:**
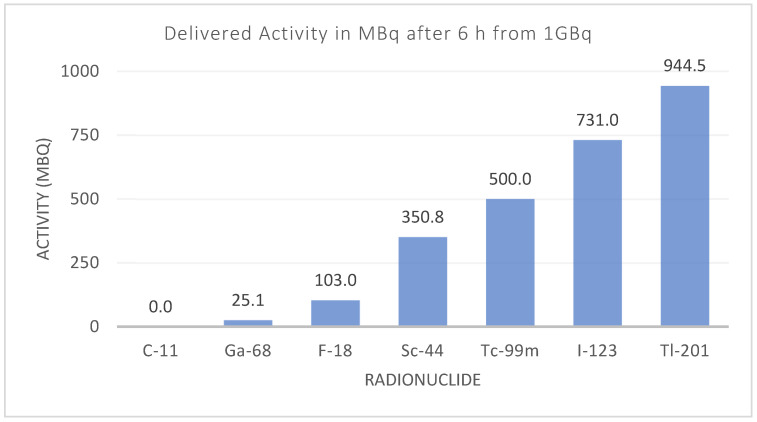
Activity for different diagnostic radionuclides six hours after production. The effect of the physical half-life can limit the radionuclide’s availability and distribution, depending on the number of facilities producing that radionuclide.

Additionally, the differences in instrumentation have also driven the introduction and reappearance of different radionuclides. Although there is increased availability of PET devices, SPECT devices are still present in large numbers (Figure 2), and they have evolved to match the quantitative capabilities of PET and pave the way towards more clinical routines using SPECT devices [59]. Other than clinical aspects, some of the main needs faced by radionuclide development include overcoming shortages in production [60,61], availability and distribution, particularly for clinics situated far away from manufacturing sites (Figure 10).

### 3.2. The Revolving World of the Radionuclide

The hunt for the “perfect” radionuclide for any given clinical indication is almost impossible. A “perfect” nuclide can be identified in combination with the chelator in question to be used for a specific application. Should one have an antibody to be applied for example, with a longer biological half-life, a radionuclide with a longer half-life will be preferred. The nuclide must be able to easily combine with the chelator and remain stable over the required period of time. Examples of a small chelator which is effective in medical application include DOTATOC and DOTATATE, which has been of particular use in the diagnosis and treatment of neuroendocrine tumours (NET). The use of Ga-68 for diagnosis and Lu-177 for therapy has ensured the popularity of these two radiometals in nuclear medicine. The ^68^Ge/^68^Ga generator was developed in the 1960s but has only recently escalated in popularity, particularly due to the development of the prostate-specific membrane antigen (PSMA). PSMA is a transmembrane glycoprotein which is overexpressed in prostate cancer cells. A number of radiolabelled PSMA probes have been developed, including the most widely-used: [^68^Ga]Ga-PSMA-11. It is well known that [^68^Ga]Ga-PSMA-11 PET/CT is superior to both conventional imaging [62] and choline-based PET/CT for evaluating prostate cancer patients, primarily in the context of biochemical failure but also for staging purposes [2,63,64,65,66,67,68,69,70,71,72,73,74]. [^18^F]F-PSMA-1007 is a novel PSMA-based radiopharmaceutical that has several advantages over [^68^Ga]Ga-PSMA-11. ^18^F-labeled agents enable large-scale production, allowing for a larger number of patient studies, as compared with the limited quantity achieved by the generator-produced Ga-68. In addition, the longer physical half-life of F-18 (T_1/2_ = 109 min) allows for its central production and distribution to satellite centres (Figure 8). F-18 also benefits from higher spatial resolution in comparison with Ga-68, due to its lower positron energy (average β^+^ energy = 250 keV), reducing the distance travelled by the positron before its annihilation and the production of the imaged gammas [75,76]. From a therapeutic perspective, Lu-177 currently holds sway as the most popular radiometal, with its ability to label to somatostatin analogues effectively working in its favour.

A possible challenge in the proposal of new radionuclides, is the effect of different biological and chemical clearances and uptakes that might be introduced. In the context of PSMA, this has been reported for unclear focal uptakes in the lymph nodes, ganglia [77] and unspecific bone uptake for F-18 [78]. The recent application of tracers that act as fibroblast-activation-protein inhibitors (FAPI) with Ga-68, again sees this radionuclide in the limelight, as it has been shown to be effective in the PET/CT imaging of 28 cancers, with fast and high tumour uptake [79].

One field that has seen many evolution steps in both radionuclide and instrumentation is nuclear cardiology. During the 1950s, the link between potassium uptake and blood flow, as well as structural and functional integrity, was demonstrated [80]. Since Tl-201 and Rb-82 have biological properties similar to potassium, they became the go-to nuclides for identifying patients with anginal chest pain and epicardial coronary artery narrowing [80,81,82]. With the arrival of Tc-99m, there was a natural shift driven by availability, instrumentation and patient exposure (Tl-201 has a much greater half-life, 73 h, compared to that of Tc-99m, 6 h) for similar quantities of injected radioactivity.

Perfusion imaging with PET is increasingly available in larger nuclear medicine departments worldwide, replacing or complementing conventional myocardial perfusion scintigraphy (MPS) in SPECT. As a result, this has seen the return of Rb-82 and the arrival of other PET tracers including [^15^O]H_2_O, [^13^N]NH_3_ and [^18^F]Fluridipaz [83]. This resurrection was only achievable with improvements in quantification accuracy, which was especially the case for Rb-82, due to its non-pure positron emissions [84,85].

## 4. Conclusions

The new developments in both instrumentation and radionuclides in nuclear medicine are dynamic fields, and their continuing evolution are intertwined. While specific radionuclides gain popularity as a result of the biological ligand being developed, it is clear that the technology used in detecting/imaging has a role to play in this too. Their combined development has propelled the growth of nuclear medicine both with respect to the used devices’ capabilities and the ever-rotating radionuclide pallet. Positron emission tomography remains the most popular mode of imaging; however, the introduction of CT, as well as the recent surge in the development of SPECT, broadens the possibilities of disease detection for physicians. As this continues to be developed, it is certain that different radionuclides will be brought in or resurrected as an attractive option for imaging, thereby ensuring the radionuclides needed in nuclear medicine do not stagnate.
